# Supplementary material for: Ultrasound Imaging of the Facial Muscles and Relevance with Botulinum Toxin Injections: A Pictorial Essay and Narrative Review
Source: Toxins (Basel). 2022 Jan 27;14(2):101. doi: 10.3390/toxins14020101 (PMC8878462; doi:10.3390/toxins14020101)

toxins

MDPI

Supplementary Materials

Ultrasound Imaging of the Facial Muscles and Relevance with Botulinum Toxin Injections: A Pictorial Essay and Narrative Review

Wei-Ting Wu, Ke-Vin Chang, Hsiang-Chi Chang, Lan-Rong Chen, Chen-Hsiang Kuan, Jung-Ting Kao, Ling-Ying Wei, Yunn-Jy Chen, Der-Sheng Han and Levent Ozcakar

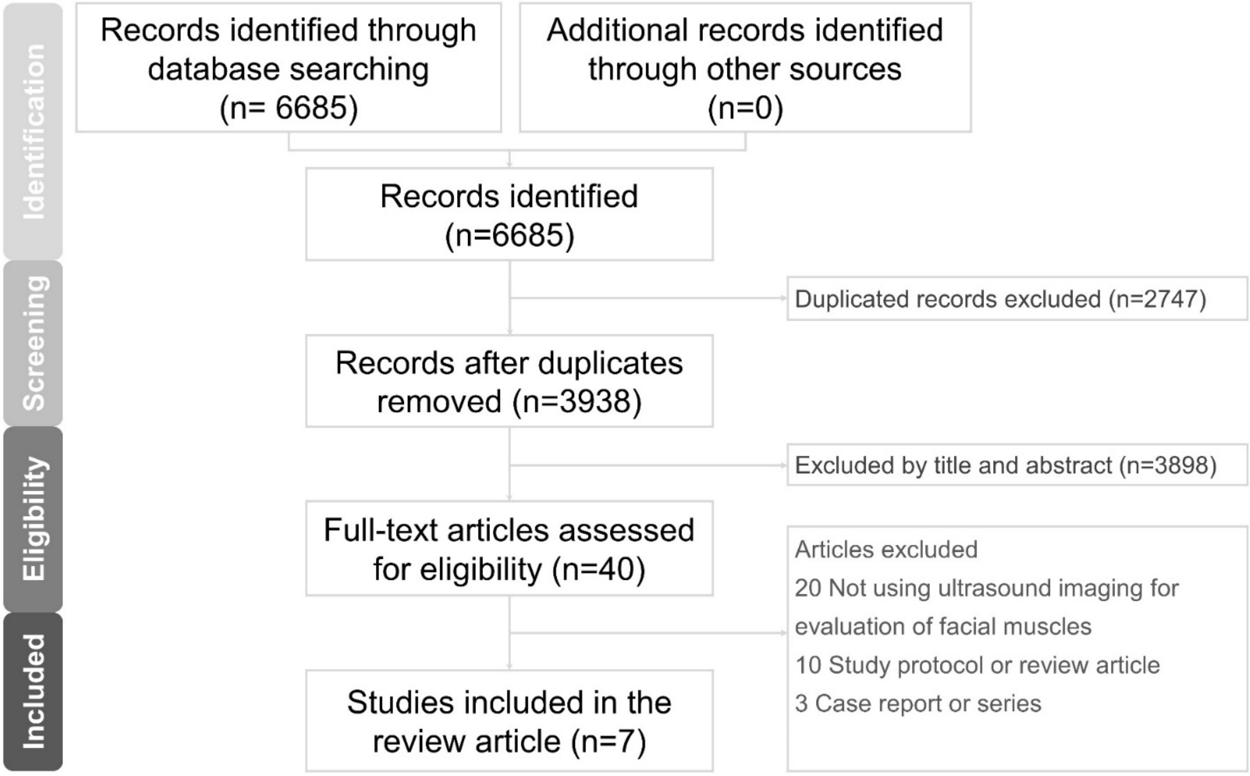

Figure S1. Flowchart of literature search.

Search results from different databases

PubMed (update on 2022/01/13)

1. (ultrasound[Title/Abstract]) OR (ultrasonography[Title/Abstract]) OR (sonography[Title/Abstract]): 380,487
2. (face[Title/Abstract]) OR (facial muscle[Title/Abstract]): 222,236
3. ((ultrasound[Title/Abstract]) OR (ultrasonography[Title/Abstract]) OR (sonography[Title/Abstract])) AND ((face[Title/Abstract]) OR (facial muscle[Title/Abstract])): 1,991

History and Search Details

Download Delete

Search	Actions	Details	Query	Results	Time
#3	•••	>	Search: **((ultrasound[Title/Abstract]) OR (ultrasonography[Title/Abstract]) OR (sonography[Title/Abstract])) AND ((face[Title/Abstract]) OR (facial muscle[Title/Abstract]))**	1,991	21:17:52
#2	•••	>	Search: **(face[Title/Abstract]) OR (facial muscle[Title/Abstract])**	222,236	21:17:19
#1	•••	>	Search: **(ultrasound[Title/Abstract]) OR (ultrasonography[Title/Abstract]) OR (sonography[Title/Abstract])**	380,487	21:16:23

Showing 1 to 3 of 3 entries

Medline (update on 2022/01/13)

1. AB ultrasound OR AB ultrasonography OR AB sonography: 341,353
2. AB face OR AB facial muscle: 233,610
3. S1 AND S2: 2,133

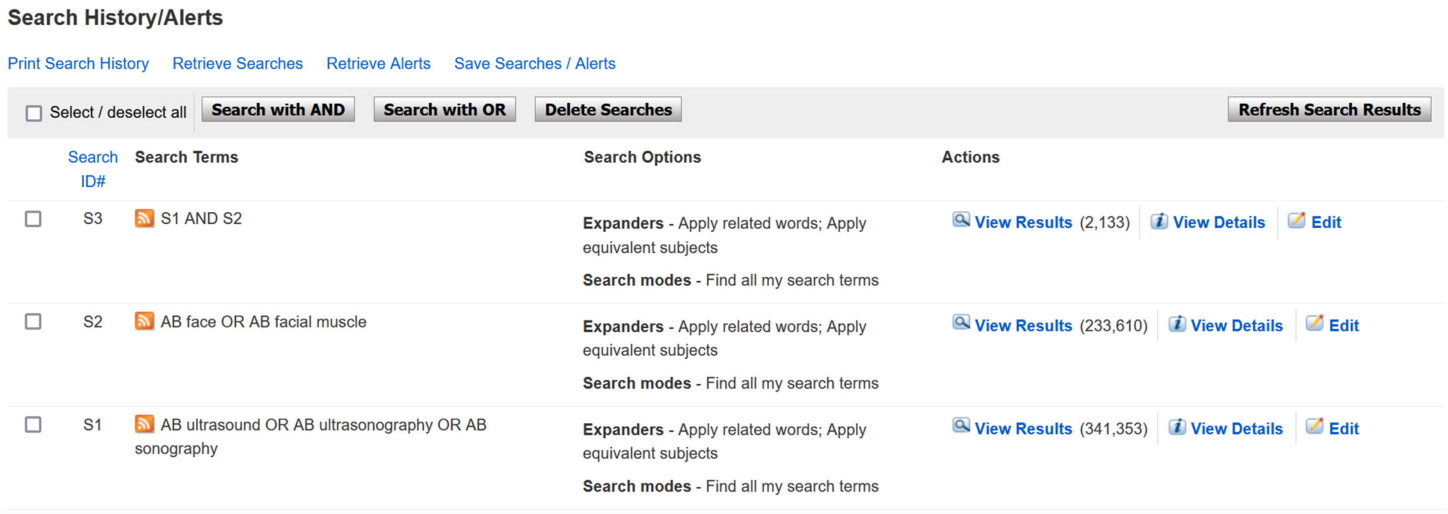
Search History/Alerts

Print Search History | Retrieve Searches | Retrieve Alerts | Save Searches / Alerts

Select / deselect all | Search with AND | Search with OR | Delete Searches | Refresh Search Results

Search ID#	Search Terms	Search Options	Actions
S3	S1 AND S2	**Expanders** - Apply related words; Apply equivalent subjects **Search modes** - Find all my search terms	View Results (2,133) View Details Edit
S2	AB face OR AB facial muscle	**Expanders** - Apply related words; Apply equivalent subjects **Search modes** - Find all my search terms	View Results (233,610) View Details Edit
S1	AB ultrasound OR AB ultrasonography OR AB sonography	**Expanders** - Apply related words; Apply equivalent subjects **Search modes** - Find all my search terms	View Results (341,353) View Details Edit

Web of science (update on 2022/01/13)

1. AB=(ultrasound) OR AB=(ultrasonography) OR AB=(sonography): 301,509
2. AB=(face) OR AB=(facial muscle): 596,940
3. #2 AND #1: 2,561

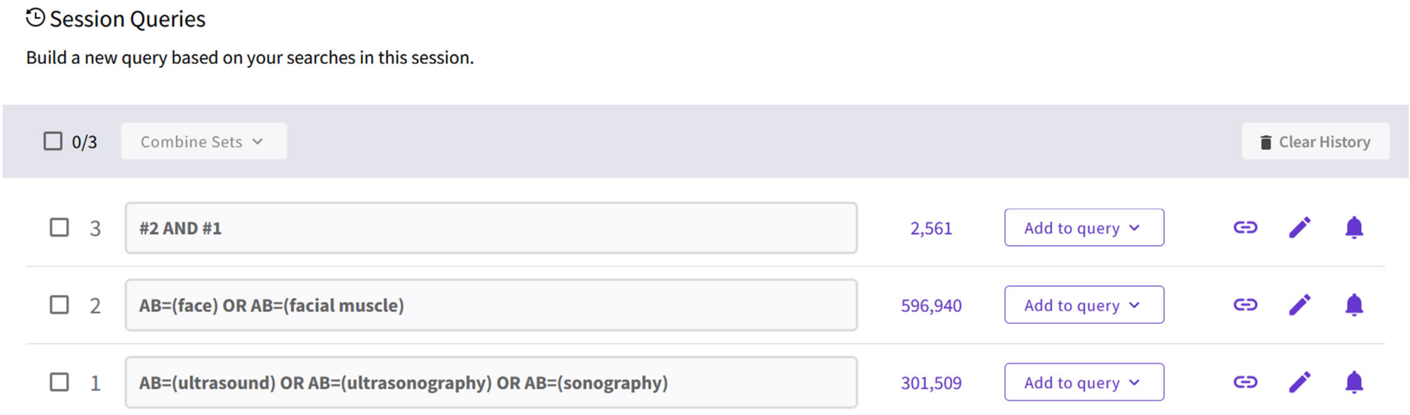

Supplement: Supplementary file 1 [file toxins-14-00101-s001.zip › toxins-1565077-supplementary.pdf]
